# Catechol-Containing Hydroxylated Biomimetic 4-Thiaflavanes as Inhibitors of Amyloid Aggregation

**DOI:** 10.3390/biomimetics2020006

**Published:** 2017-05-09

**Authors:** Matteo Ramazzotti, Paolo Paoli, Bruno Tiribilli, Caterina Viglianisi, Stefano Menichetti, Donatella Degl’innocenti

**Affiliations:** 1 Dipartimento di Scienze Biomediche, Sperimentali e Cliniche, Università degli Studi di Firenze, viale G.B. Morgagni 50, 50134 Firenze, Italy; paolo.paoli@unifi.it (P.P.); donatella.deglinnocenti@unifi.it (D.D.); 2 Dipartimento di Chimica “Ugo Schiff”, Polo Scientifico e Tecnologico, Università degli Studi di Firenze, via della Lastruccia 3-13, 50019 Sesto Fiorentino, Firenze, Italy; caterina.viglianisi@unifi.it; 3 Consiglio Nazionale delle Ricerche (CNR), Istituto dei Sistemi Complessi, Via Madonna del Piano, 10, 50019 Sesto Fiorentino, Firenze, Italy; bruno.tiribilli@isc.cnr.it

**Keywords:** catechol, hydroxylated 4-thiaflavanes, inhibition, amyloid aggregation, hen egg white lysozyme, antioxidant activity

## Abstract

The study of compounds able to interfere in various ways with amyloid aggregation is of paramount importance in amyloid research. Molecules characterized by a 4-thiaflavane skeleton have received great attention in chemical, medicinal, and pharmaceutical research. Such molecules, especially polyhydroxylated 4-thiaflavanes, can be considered as structural mimickers of several natural polyphenols that have been previously demonstrated to bind and impair amyloid fibril formation. In this work, we tested five different 4-thiaflavanes on the hen egg-white lysozyme (HEWL) amyloid model for their potential anti-amyloid properties. By combining a thioflavin T assay, atomic force microscopy, and a cell toxicity assay, we demonstrated that such compounds can impair the formation of high-order amyloid aggregates and mature fibrils. Despite this, the tested 4-thiaflavanes, although non-toxic per se, are not able to prevent amyloid toxicity on human neuroblastoma cells. Rather, they proved to block early aggregates in a stable, toxic conformation. Accordingly, 4-thiaflavanes can be proposed for further studies aimed at identifying blocking agents for the study of toxicity mechanisms of amyloid aggregation.

## 1. Introduction

Amyloid aggregation is a degenerative process characterized by deposition at tissue levels of organized insoluble super-molecular protein assemblies with a typical cross-β secondary structure. Such degeneration gives rise to amyloidosis, a composite range of diseases classically divided into neurodegenerative (e.g., Alzheimer’s disease, Parkinson’s disease, etc.) and systemic (e.g., cystic fibrosis, light chain amyloidosis) amyloidosis. More than 20 different human proteins, intact or fragmented, proved their amyloidogenicity in vivo, among which we may count amyloid β (Aβ) peptide (in Alzheimer’s disease), α-synuclein (in Parkinson’s disease), islet amyloid polypeptide (in type II-diabetes), light chains of immunoglobulins, variants of human lysozyme [1,2], and transthyretin (TTR) [3]. It is nowadays widely accepted that amyloid aggregation is a general tendency of polypeptide chains [4,5,6] that, in fact, may be induced to form amyloid aggregation in appropriate conditions [7].

Lysozyme, a 130-residue-long bacteriolytic enzyme largely distributed in different tissues, organs, and external secretions, has been highlighted as an interesting model for the study of amyloid aggregation. Although wild-type lysozyme is not directly involved in amyloid diseases, several naturally occurring single point mutations (e.g., Ile56Thr, Phe57Ile, Trp64Arg, and Asp67His) are connected with familial non-neuropathic systemic amyloidosis [8]. In addition, the wild-type lysozyme either from humans, horses, or hens, under appropriate conditions, is able to form amyloid fibrils in vitro [9,10,11].

In this work, we used the hen egg-white lysozyme (HEWL—14.3 kDa, 129 amino acids, 40% identity with the human enzyme) inducing its aggregation through a heat treatment in acidic conditions [12]. Despite the fact that HEWL is not associated with in vivo diseases [13], it has been demonstrated that high temperatures and low pH induce the breakage of X-Asp peptide bonds, leading to the formation of peptide fragments (among which one contains the residues corresponding to those mutated in human familiar diseases Ile56Thr and Asp67His). Such fragments have a high tendency to form amyloid aggregates [11] and amyloid-like fibrils in a few days. In addition, a direct toxic effect of HEWL aggregates added to cell cultures or injected in rat brains, mimicking the toxic effect of Aβ peptide, has been demonstrated [14].

A substantial body of literature over the years documents that extracts from natural herbs and plants or common dietary elements such as wine [15] or green-tea [16] are of great benefit to general human health, mainly due to their antioxidant power [17,18]. Among the molecules proposed to be essential for achieving such effects are polyphenols [19], a wide and heterogeneous group of substances well characterized in terms of structure [20]. Apart from protecting cells from oxidative stress, several phenolic compounds have been shown to be effective in inhibiting amyloid aggregation in various protein models such as transthyretin, microglobulin, α-syuclein, Aβ peptide and lysozyme, with supposed specific action mechanisms independent of their antioxidant properties [21,22,23,24,25]. Of particular relevance for this work, catecholic- and hydroquinone-containing phenols were reported to act as inhibitors of amyloid aggregation for their ability to induce quinoprotein formation [26].

**Figure 1:**
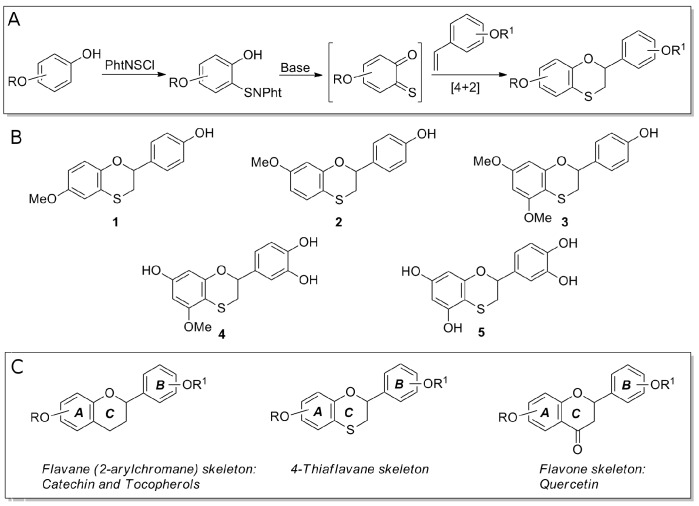
Design of hydroxylated 4-thiaflavanes and their biomimetisms. (**A**) Synthesis of hydroxylated 4-thiaflavanes used in this study. (**B**) Structure of 4-thiaflavanes tested in this study. (**C**) Biomimetism of 4-thiaflavanes with 2-arylchromane (flavane) and flavone skeletons.

Dihydrobenzo[1,4]oxathines, and in particular compounds possessing a polyhydroxylated 4-thiaflavane skeleton, have received great attention in chemical, medicinal, and pharmaceutical research. During the last decades, their syntheses as well as their abilities as antioxidants, hypertensive agents, estrogen receptor modulators, adrenoreceptor antagonists, and artificial sweeteners have been reported in papers and patents [27]. As shown in Figure 1, such 4-thiaflavane derivatives are structural mimickers of several natural polyphenols. In particular, compounds **4** and **5** (see Figure 1) are thia-substituted biomimetic examples of catechin derivatives showing a free catechol residue on the B ring. In this work, we investigated whether selected hydroxylated 4-thiaflavane derivatives may act as an inhibitor of amyloid aggregation for the widely used and accepted HEWL amyloid model.

## 2. Materials and Methods

### 2.1. Materials

Reagents and chemicals, unless otherwise specified, were purchased from Sigma-Aldrich (St. Louis, MO, USA) and used without further purification, including lysozyme (HEWL, code L6876).

### 2.2. Preparation of 4-Thiaflavane Derivatives

Hydroxylated 4-thiflavanes were prepared as previously reported by inverse electron demand hetero Diels–Alder reaction of transient *ortho*-thioquinones with properly substituted styrenes. This allowed for the direct synthesis of compounds bearing hydroxy or methoxy groups on the selected position of the A and/or B ring of the thiaflavane skeleton (Figure 1) [28,29,30].

**Figure 2:**
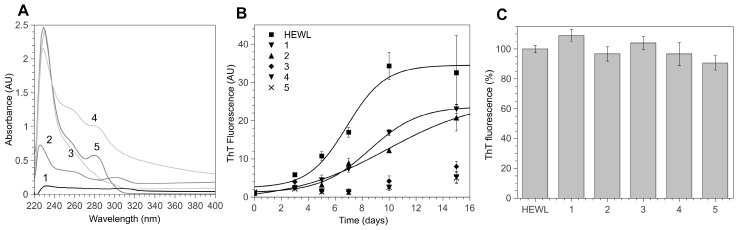
4-Thiaflavane derivatives inhibit hen egg-white lysozyme (HEWL) amyloid aggregation. (**A**) Absorption spectra of 4-thiaflavanes derivatives (compound **1**–**5**) used in this study. (**B**) Aggregation kinetic of HEWL in the presence of 4-thiaflavane compounds as followed by a thioflavin T (ThT) assay for 15 days. (**C**) The absence of competition of 4-thiaflavane compounds with ThT on preformed HEWL fibrils. Values in (**B**,**C**) are expressed as the mean ± standard deviation of at least three independent measurements. AU: Arbitrary units.

Lyophilized thiaflavane powders were weighted and dissolved in pure dimethyl sulfoxide (DMSO) at a concentration of 100 mM. For assays, they were freshly diluted at appropriate concentrations in assay buffers. The chemical structures of the thiaflavanes used in this study are depicted in Figure 1 and their absorbance spectra shown in Figure 2A.

### 2.3. Preparation of Hen-Egg White Lysozyme for Aggregation

HEWL solutions were freshly prepared before each assay. HEWL powder was weighted and dissolved in 10 mM HCl (pH 2, HEWL buffer) via vortexing at room temperature at a concentration of 1 mM (14.7 mg/mL). The solution was then filtered using 0.2 µm syringe filter discs (EMD Millipore, Milan, Italy).

### 2.4. Aggregation Conditions

HEWL aggregation was achieved by incubating the pH 2 solution at 65 °C for up to 15 days [11,31]. 4-Thiaflavanes were mixed to HEWL at a 1:1 molar ratio at a 1 mM concentration, immediately before incubation at 65 °C. Control HEWL aggregation was performed in the presence of 1% DMSO. All aggregation experiments were repeated at least three times.

### 2.5. Thioflavin T Assay

For the thioflavin T (ThT) assay [32], 25 µM ThT was freshly prepared from a 2.5 mM stock solution in a 25 mM sodium phosphate buffer, pH 6 (ThT buffer). For each measurement, 245 µL of the diluted ThT solution was added to a sample volume of 5 µL on a polystyrene multiwell plate. ThT fluorescence was measured at 25 °C with a Fluoroskan Ascent FL multiwell plate reader (Thermo Scientific, Waltham, MA, USA) using 440 nm and 485 nm as excitation and emission wavelengths, respectively. All measurements were performed in triplicate. Kinetic traces were analyzed via non-linear fitting to the sigmoidal function *F* = *F_f_* + (*F_i_* − *F_f_*)/(1 + exp((*x* − *t_0_*)/*d_x_*)), *F* being the time-dependent fluorescence intensity, *F_i_* the fluorescence at the beginning of the aggregation, *F_f_* the fluorescence at the end of the aggregation process, *t_0_* the time at which 50% of the total variation in fluorescence is reached, and *d_x_* the time constant. The apparent rate constant (*kf*) for the growth of fibrils is given by 1/*d_x_*, the lag time is calculated as *t_0_* − 2*d_x_*, and the fluorescence amplitude is given by *Ff* − *Fi*. All these analyses were performed with QtiPlot v0.9.8.0 software (http://www.qtiplot.com).

### 2.6. Atomic Force Microscopy

For atomic force microscopy (AFM) analysis, a drop of aggregating solutions (HEWL with or without 4-thiaflavanes) were vortexed and laid onto a freshly cleaved mica disc (Ted Pella Inc., Redding, CA) for about 2 min. Excess of sample was removed by washing twice with 1 mL of bidistilled water, the preparation was then dried with a soft nitrogen flow. AFM experiments were performed in air, in non-contact mode, using a PicoSPM microscope equipped with an AAC-Mode controller (Molecular Imaging, Phoenix, AZ, USA). The probes were non-contact Silicon cantilevers (model NSG-01, NT-MDT Co., Moscow, Russia) with a 150 KHz typical resonance frequency. Scanner calibration was periodically checked by means of a reference grid (TGZ02 by MikroMash, Tallin, Estonia) with a known pitch of 3 μm and a step height of 100 nm. Scan size ranged from 450 × 450 nm to 30 × 30 µm. Images were processed and analyzed with Gwyddion software v2.34 (http://gwyddion.net). For the analysis, the pre-processing involved (i) levelling the map by mean plane subtraction, (ii) correcting lines by matching height median, (iii) correcting horizontal artefacts (scars), (iv) applying a Gaussian smoothing filter of 2 px, and (v) shifting minimum data value to zero.

### 2.7. Cell Growth and Citotoxicity Assay

Human SH-SY5Y neuroblastoma cells (American Type Culture Collection, Manassas, VA, USA) were cultured in Dulbecco's modified Eagle's medium DMEM F-12 Ham with 25 mM HEPES (N-2-hydroxyethylpiperazine-N-2-ethane sulfonic acid) and NaHCO3 (1:1) supplemented with 10% fetal bovine serum (FBS, Sigma-Aldrich), 1 mM glutamine, and antibiotics.

The cytotoxicity of the aggregates was assessed by an MTT (3-(4,5-dimethylthiazol-2-yl)-2,5-diphenyltetrazolium bromide) reduction inhibition assay [33]. Briefly, SHSY-5Y cells in exponential growth were incubated for 48 h in the presence of HEWL aggregates matured alone or in the presence of 4-thiaflavanes. The growth medium was removed, and the plates were incubated for 2 h in a 5% CO_2_-humidified atmosphere at 37 °C in the presence of a medium solution containing 0.5 mg/mL of the MTT reagent. After 2 h, the solution was removed and replaced with a lysis buffer containing 20% sodium dodecyl sulfate (SDS) and 50% dimethylformamide (DMF, pH 4.7), and further incubated for 1 h. The absorbance of blue formazan was measured at 570 nm with an iMark^TM^ microplate reader (BioRad, Hercules, CA, USA).

## 3. Results

### 3.1. 4-Thiaflavane Derivatives Obstacle/Impair Amyloid Aggregation Kinetics

HEWL aggregation is primed by a fragmentation process that may be induced by heating a concentrated HEWL solution in acidic conditions. We tested the anti-aggregation properties of hydroxylated 4-thiaflavanes (Figure 1) on HEWL by incubating them at a 1:1 molar ratio prior to heating, thus allowing the fragmentation to occur in the presence of 4-thiaflavanes under study. The aggregation kinetic of HEWL in the presence of 4-thiaflavanes was followed by ThT assay (a universally accepted fluorogenic probe for cross β-aggregates). HEWL aggregation proved to be deeply altered by three out of the five 4-thiaflavanes tested, namely **4**, **5**, both containing a catechol residue, and **3** (Figure 2B). While compounds **3**, **4** and **5** were able to almost completely inhibit ThT signals, suggesting a deep impact on amyloid aggregate maturation, compounds **1** and **2** proved to have little effect on the estimated lag phase of the aggregation process (3 ± 2 and 5 ± 0.5 days for **1** and **2**, respectively, compared to 4 ± 1 days for HEWL) and a moderate effect on the plateau phase (about 30%). This analysis suggested that aggregation is not completely abolished and possibly stabilized in a different final conformation, with lower affinity for the ThT dye or with a lower concentration of amyloid aggregates.

### 3.2. 4-Thiaflavane Derivatives Do Not Compete with Thioflavin T

It has been recently demonstrated that ThT signals can be strongly affected by compounds with phenolic moieties, due to the optical or physical competition with the ThT binding site on aggregates [34], leading to misinterpretation of amyloid inhibition by exogenous compounds. In order to exclude optical interferences, we measured the absorption spectra of 4-thiaflavane derivatives dissolved at a 1 mM concentration in a ThT buffer using 1% DMSO as a blank. Figure 2A shows that none of the substances tested showed significant absorption in the 400–500 nm region, where ThT is excited and emitted when cross-β structures are present. Secondly, we tested whether 4-thiaflavanes could compete with ThT by incubating them with ThT on preformed HEWL amyloid fibrils. The rationale behind this assay was based on the assay time: amyloid aggregates are considered extremely stable and resistant to disaggregation, being solubilized only by strong denaturing agents such as DMSO or hexafluoroisopropanol (HFIP). It therefore appears unrealistic that disaggregation could occur in a few minutes of assay time in very mild conditions such as those required for the ThT assay. As shown in Figure 2C, none of the 4-thiaflavanes proved to compete significantly with ThT, since fluorescence signals recorded in the presence of preformed fibrils were not reduced by the addition of 4-thiaflavanes at concentrations ten times higher than that of ThT. We therefore concluded that competition or interference could not have been the reason for the fluorescence loss in our kinetic experiments.

### 3.3. Polyhydroxylated 4-Thiaflavanes Inhibit the Formation of Amyloid Fibrils

**Figure 3:**
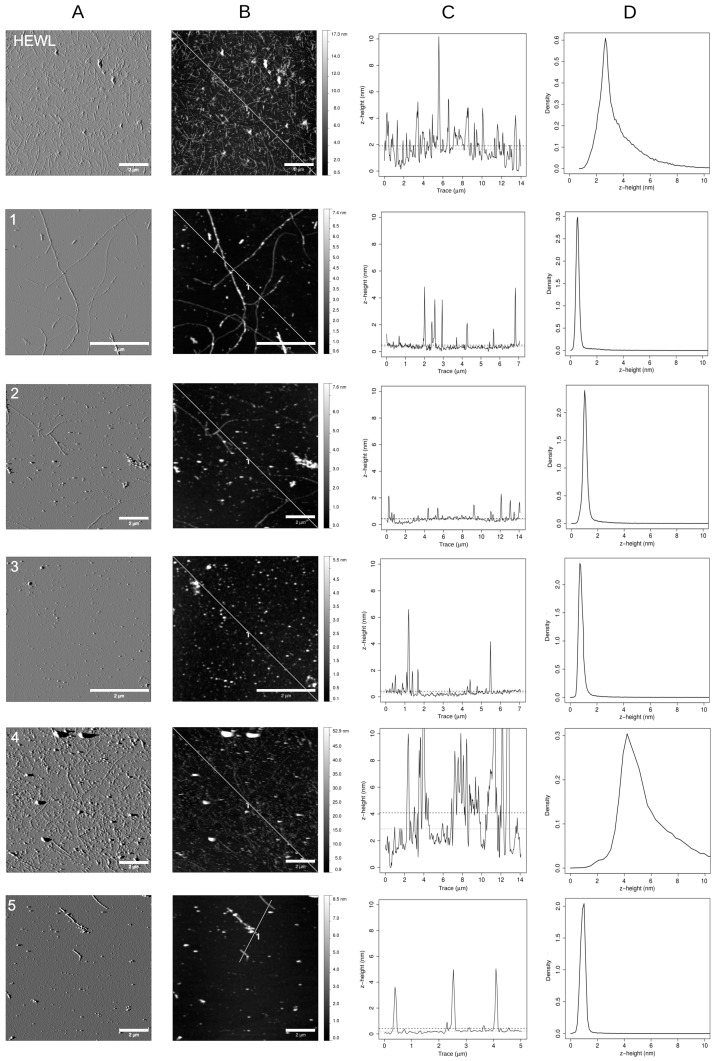
Atomic force microscopy (AFM) maps of HEWL incubated in aggregation conditions for 10 days in the presence or absence of the 4-thiaflavane derivatives (compounds **1**–**5**): (**A**) amplitude maps; (**B**) morphology maps and the corresponding (**C**) profile traces, together with the mean and median *z*-high values plotted as horizontal dashed or dotted lines, respectively; (**D**) height distribution of the whole map. Scale bar: 2 µm.

In order to study the morphology of the HEWL amyloid aggregates after 10 days (the time required to HEWL to convert into mature fibrils), we deposited the aggregating solutions on freshly cleaved mica and its surface was scanned by AFM. As shown in Figure 3, HEWL fibrils with a height of about 4 nm were highly abundant in the absence of 4-thiaflavane derivatives. Their burden was found to be highly reduced in the presence of all 4-thiaflavane derivatives.

By combining morphology maps, height distribution analysis, and *z*-profile analysis, we studied the morphology of aggregates formed at this time point. Compound **1** showed a drastic reduction in the number of fibrils formed and the accumulation of round particles of about 2–4 nm in height, a size similar to the diameter of the residual fibrils. A similar behavior was observed for compound **2** with an apparent increase in the ability of reducing fibrillar structures. Both compounds were only in part effective in reducing ThT signals, suggesting that round particles partly maintain the cross-β amyloid structure targeted by such dye, resembling the structure of early aggregates. Compound **3** proved to completely abolish the formation of fibrils, resulting in the accumulation of round particles again with an apparent size of 2–4 nm but with a drastically reduced affinity for ThT. The aggregation of HEWL in the presence of compound **4** exhibited a completely different morphological pattern, showing a mixture of round particles of different diameters and small fibrils. The fibrils observed in this case were almost invariantly larger than that observed with other compounds or untreated HEWL, with apparent diameters above 10 nm, and unable to give rise to an increase in ThT signals. In addition, and peculiar to this compound, particles with heights surpassing 100 nm were detected. A complete absence of fibrils and a drastic reduction in globular particles of 3–5 nm were eventually observed for compound **5**. Details of the results of the *z*-profile measurements for all samples are further available as accompanying supplementary materials.

### 3.4. 4-Thiaflavane Derivatives Do Not Prevent Toxicity Induced by Early Amyloid Aggregates

**Figure 4:**
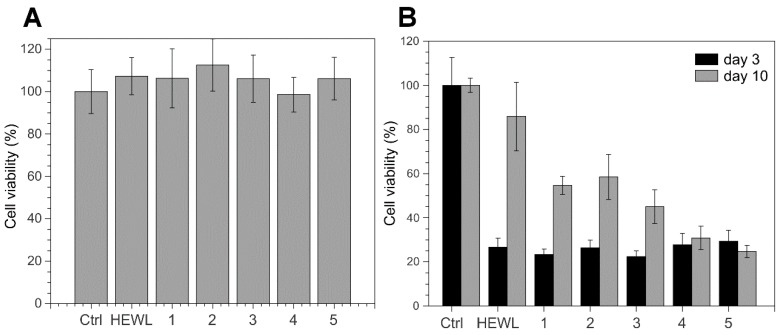
Cytotoxicity assay on SHSY5Y cells. (**A**) Absence of cell toxicity after 48 h incubation with monomeric HEWL or 4-thiaflavanes (compounds **1**–**5**). (**B**) Cell toxicity after 48 h incubation with 3 and 10 days old aggregates of HEWL incubated with or without 4-thiaflavanes (compounds **1**–**5**). Bars represent the mean ± standard deviation of at least three independent measurements. Ctrl: untreated cells.

For assessing whether the drastic reduction of aggregate load evidenced in both ThT assay and AFM was accompanied by a reduction of aggregate-induced cytotoxicity, we incubated actively growing SHSY-5Y neuroblastoma cells with HEWL aggregates that had matured for 3 days both alone or in the presence of thiaflavanes. In fact, in a preliminary cytotoxicity kinetic analysis, the 3-day incubation time proved to be the most toxic phase of HEWL aggregates formed in our conditions (data not shown). 4-Thiaflavane derivatives alone proved to be safe for cells at concentrations used in the assay (Figure 4A). On the contrary, we found that 4-thiaflavanes were able to show little or no protective effects on cells treated with 3-day aggregates (Figure 4B), indicating that premature toxic aggregates were present in the solutions and that their formation was not prevented by the presence, during the aggregation process, of such compounds. This is in clear contrast to the impairment of the polymerization process evidenced by ThT and by AFM. In order to exclude possible artefacts due to the heat-induced conversion into toxic compounds of 4-thiaflavanes tested, we also heated compounds alone. We verified that 4-thiaflavanes were also safe for cells after heating, resulting in cells that showed no signs of toxicity. We also verified the opposite condition, i.e., that heating could have induced a loss of protective effect that 4-thiaflavane derivatives could have exerted per se (e.g., as an antioxidant, or with some other property independent of the effect of amyloid material). To do this, we added freshly prepared 4-thiaflavanes to cells, concomitantly with untreated 3-day aggregates. Additionally, in this case, we did not find particular signs of protection. When the cells were treated with aggregates that had matured for 10 days, a completely different picture was observed. HEWL alone, in the form of mature fibrillar aggregates (see Figure 3), showed a markedly reduced toxicity with respect to the untreated control. On the contrary, thiaflavane-treated HEWL showed a pronounced toxicity, similar to that observed for the 3-day maturation especially for compounds **4** and **5** (Figure 4B).

## 4. Discussion

The possibility of blocking or reverting amyloid aggregation with small molecules may have a great impact on worldwide health. Amyloidosis, a group of over 20 different and heterogeneous diseases, are directly linked to the accumulation of amyloid matter into organs and are severely affecting human population. Amyloidosis, including neurodegenerative diseases such as Alzheimer’s disease or Parkinson’s disease as well as systemic diseases such as reactive systemic amyloidosis, TTR, and light chain amyloidosis are increasingly recognized as important death factors for public health systems.

Many studies have so far demonstrated that molecules with peculiar structural features are able to impair the formation or the elongation of amyloid fibrils, among which are a number of synthetic or natural compounds with polyphenolic rings [23]. Several activity–structure studies have shown that, though the effect of the addition of such exogenous compounds could be beneficial in some cases in terms of the prevention of amyloid formation or the reduction of amyloid load, the activity spectrum is bound to a defined experimental set and confined to certain amyloid related proteins and peptides, such as Aβ peptides, α-synuclein, and TTR.

In this study, we used HEWL as a model of amyloid aggregation. The reason for this choice was based on the need to decouple the effect of metal ions on aggregation to the documented chelating properties of 4-thiaflavanes [35]. In fact, many amyloid models have been found to be deeply affected by the presence of copper or iron ions, and in traces, leading to alterations of the aggregation kinetics, of the lag phase, or of the morphology of the resulting fibrils. Such effects have never been reported for HEWL, so we considered it a good model for our class of compounds, allowing us to establish a direct link between the structure of the molecules and the effect on aggregation inhibition. In fact, we found that the poor solubility of compounds such as **1** and **2** is not of benefit in reducing amyloid signals, which is contrary to what has been found for other compounds, such as curcumin, which has been declared to be extremely active in aggregation in vitro despite its absolute insolubility in aqueous buffers. In our conditions, the most active 4-thiaflavanes were molecules with a higher degree of hydroxylation, leading to increased solubility and a reasonably higher capability of interacting with nascent aggregates, blocking their elongation into higher order assemblies such as mature fibrils. Moreover, despite the lack of a direct relationship between antioxidant activity and the inhibition of amyloid aggregation, it is worth mentioning that compounds **4** and **5**, containing a catechol moiety, showed a much higher antioxidant activity with respect to compounds **1**, **2**, and **3** [36].

A relevant body of literature exists trying to shed light on the true nature of the cytotoxicity induced by amyloid material. Initially, it was strongly believed that toxicity was due to early aggregates only (a stage at which large portions of hydrophobic protein regions are exposed, waiting to gain stabilization, with polymerization and burial of these regions inside highly ordered structures). Recently, it has been shown that this cannot be considered a general rule because of the existence of proteins for which mature fibrils largely surpass the toxicity of early aggregates [37]. HEWL has been previously shown to behave in the “classic” way, losing toxicity as fibrils grow and as early aggregates are progressively sequestered from the medium to the fibril. We verified this behavior in our experimental conditions, selecting day 3 as the one displaying the maximal toxicity that was progressively lost at longer times, until day 10, when fibrils were completely mature, as shown by AFM. At this time point, we measured extremely reduced ThT signals for compounds **3**, **4**, and **5**, but we found a toxicity comparable to that of untreated HEWL. When the incubation time was increased to allow HEWL controls to develop into non-toxic mature fibrils, the HEWL samples treated with active compounds were found to maintain a toxicity comparable to samples tested at early aggregation stages. Nevertheless, the ThT signal proved to be, in most cases, highly reduced. Our results seem to suggest that the effect of 4-thiaflavane derivatives was to impair (in part or almost completely, depending on the compound) the elongation of HEWL fibrils, leading to the formation of round particles with diameters similar to that of amyloid species and that are highly toxic to cells, stable over time, and poorly responsive to ThT binding.

According to a recent finding, molecules containing catecholic and (less efficiently) hydroquinone moieties may be able to drive the formation of quinoproteins, i.e., to covalently modify proteins in a hot acidic environment [26] and hamper HEWL fibril formation. Compounds **4** and **5** bear in the B ring a catechol-like structure that, given aggregation conditions (acidic pH and high temperature), may induce protein derivatization and form *ortho*-quinonic adducts. Similarly, compounds **1**, **2**, and **3** present a phenol ring that, in the same conditions, can undergo acid hydrolysis and further oxidation, leading to the formation of p-quinoic adducts. Our results on hydroxylated 4-thiaflavanes seem to suggest that the abovementioned reaction mechanisms may drive the observed reduction in amyloid formation without a loss of cell toxicity. The ability of compound **3** to drastically reduce the load in fibrils (despite its structural similarity with the less effective compounds **1** and **2**) partly contrasts this vision and suggests that additional research is required to definitively validate the above hypothesis.

Although our findings strongly discourage the usage of the investigated 4-thiaflavanes as potential drugs for amyloidosis, the opposite route seems interesting. Since these compounds have been shown to be safe for cells at the tested doses, their contribution to cell toxicity following incubation with toxic aggregates is minimal, allowing a coherent assay of aggregate toxicity. In fact, one of the most challenging aspects of amyloid studies is that intermediate, toxic structures are transient in nature and their effect is confined in a restricted, highly variable temporal frame.

Further biophysical studies will be needed to elucidate the structures and the features of the toxic aggregates stabilized by the 4-thiaflavane derivatives tested in this work and their mechanism of action. Of particular interest is the observation that such particles give reduced or impaired ThT signals, a fact that is counterintuitive given their toxicity, which is similar to 3-day HEWL early aggregates, which proved to give rise to aggregates that efficiently bind ThT. Furthermore, it cannot be excluded that the structural features of such aggregates may be different in response to different compounds, although the results collected so far seem to suggest as the major player a population of small, globular particles with a quite uniform size.

If confirmed on other amyloid systems, these results suggest that hydroxylated 4-thiaflavanes are promising stabilizing agents for toxic aggregates that are useful for studying, for example, the effect of single or multiple mutations on a uniform and homogeneous population of toxic species.
